# Switchable Quantized Signal between Longitudinal Conductance and Hall Conductance in Dual Quantum Spin Hall Insulator TaIrTe_4_

**DOI:** 10.34133/research.0439

**Published:** 2024-08-22

**Authors:** Junwen Lai, Xiangyang Liu, Jie Zhan, Tianye Yu, Peitao Liu, Xing-Qiu Chen, Yan Sun

**Affiliations:** ^1^ Shenyang National Laboratory for Materials Science, Institute of Metal Research, Chinese Academy of Sciences, Shenyang 110016, China.; ^2^ School of Materials Science and Engineering, University of Science and Technology of China, Shenyang 110016, China.

## Abstract

Topological insulating states in 2-dimensional (2D) materials are ideal systems to study different types of quantized response signals due to their in gap metallic states. Very recently, the quantum spin Hall effect was discovered in monolayer TaIrTe_4_ via the observation of quantized longitudinal conductance that rarely exists in other 2D topological insulators. The nontrivial *Z*_2_ topological charges can exist at both charge neutrality point and the van Hove singularity point with correlation-effect-induced bandgap. On the basis of this model 2D material, we studied the switch of quantized signals between longitudinal conductance and transversal Hall conductance via tuning external magnetic field. In *Z*_2_ topological phase of monolayer TaIrTe_4_, the zero Chern number can be understood as 1 – 1 = 0 from the double band inversion from spin-up and spin-down channels. After applying a magnetic field perpendicular to the plane, the Zeeman split changes the band order for one branch of the band inversion from spin-up and spin-down channels, along with a sign charge of the Berry phase. Then, the net Chern number of 1 – 1 = 0 is tuned to 1 + 1 = 2 or −1 – 1 = −2 depending on the orientation of the magnetic field. The quantized signal not only provides another effective method for the verification of topological state in monolayer TaIrTe_4_ but also offers a strategy for the utilization of the new quantum topological states based on switchable quantized responses.

## Introduction

Topological states in 2-dimensional (2D) systems have been extensively studied in the past decades. The quantum Hall (QH) effect is the first discovered topological state in materials, which presents as quantized Hall conductance in the unit of *e*^2^/*h* with zero longitudinal conductance [1–4]. The quantized Hall conductance originated from the nondissipation chiral edge state, while all the other states are localized. In electronic band structures, the occupied and nonoccupied states are connected by the chiral edge states located in the bulk bandgap. The number of net edge channels can be understood from the Chern number of bulk band structures. Since the Hall conductance in the QH effect is only dependent on the fundamental constant of the electron charge and the Planck constant, it plays an important role in the metrology resistance standards and quantum computing [5–8].

In history, most of the QH effect states were measured in 2D electron gas under strong perpendicular magnetic fields [9–14]. With the generalization of topological band theory in condensed matter physics, it is found that different types of topological states exist in nature and applied magnetic fields can control different topological phase transitions, including both insulating and semimetal states. With this guiding principle, the QH effect and quantum anomalous Hall effect were realized in topological insulators and the thin film of Dirac semimetal, [15–28] and the QH effect is even generalized into 3D electron systems [29–33].

The interplay between the magnetic field and 2D topological materials provides an ideal platform for the study of topological phase transition among quantum spin Hall (QSH) insulators, topological semimetal, QH effect, and quantum anomalous Hall effect [34–36]. In addition to plenty of quantum topological phases, the topological phase transition also offers an effective approach for detecting the topological states from quantized transport signals. Very recently, the theoretic proposed dual QSH insulator state of TaIrTe_4_ monolayer was confirmed by the experimentally fabrication [37–39]. The nontrivial *Z*_2_ topological charges in TaIrTe_4_ exist at both the charge neutrality point and the van Hove singularity point with a new bandgap induced by strong correlations. Because of its 2D nature, monolayer TaIrTe_4_ can be understood as a model material for realizing different types of topological states under perturbations of strain, gating, magnetic field, and magnetic doping.

**Fig. 1.**
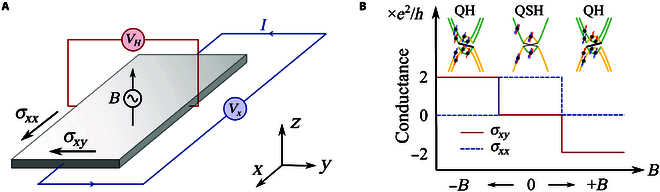
Schematic illustration of the magnetic field-induced conductance change under topological phase transition. (A) Illustration of the longitude(*σ_xx_*) and Hall(*σ_xy_*) conductance under a varying perpendicular magnetic field *B*; the input current *I* is along the *x* direction. (B) Topological-phase-transition-induced conductance (*σ_xx_*, *σ_xy_*) change under a varying perpendicular magnetic field *B*, the yellow and green colors stand for the different orbitals, and the red and blue arrows on the band structure represents the spin-up and spin-down states, respectively. The magnetic field is simulated by an out-of plane Zeeman field here.

In this work, we studied the evolution of magnetic-field-induced topological phase transition from *Z*_2_ QSH insulator to QH insulator. Along with these phase transitions, the quantized signals of longitudinal conductance and Hall conductance are switched on and off via the control of an external magnetic field, as schematic shown in Fig. 1. The topological phase transition provides an effective approach to obtaining a QH effect state starting from the time-reversal symmetry 2D topological insulators, and the switchable quantized signals offer a strategy for the utilization of the new quantum topological material.

## Results and Discussion

**Fig. 2.**
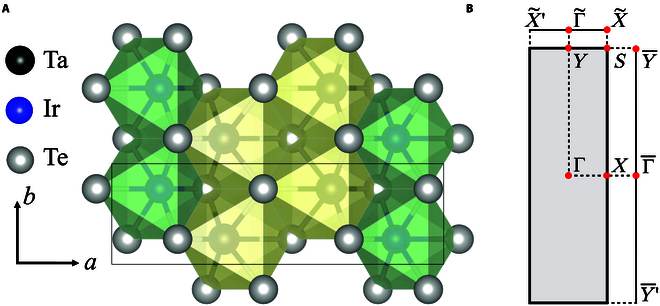
The crystal structure and BZ of the monolayer TaIrTe_4_. (A) Top view of the monolayer TaIrTe_4_. (B) BZ and its projection to different direction [100] and [010] of the monolayer TaIrTe_4_.

Bulk TaIrTe_4_ is a well-known type II Weyl semimetal that crystallized with an AB stacking of 2 centrosymmetric van der Waals layers [40,41]. Its monolayer was theoretically predicted as a QSH insulator at the charge neutrality point and very recently experimentally verified by the observation of quantized longitudinal conductance [37–39]. In addition, nontrivial *Z*_2_ bandgaps of a correlated charge density wave (CDW) state were also observed by shifting Fermi level to the van Hove singularity points [37–39] via weakly doping. The monolayer TaIrTe_4_ crystallized in a space group of *P*2_1_/*m* (no. 11), which consists of 2 symmetry operators of inversion *i* and {*C*_2*y*_ ∣ (0, 1/2, 0)}, as shown in Fig. 2.

**Fig. 3.**
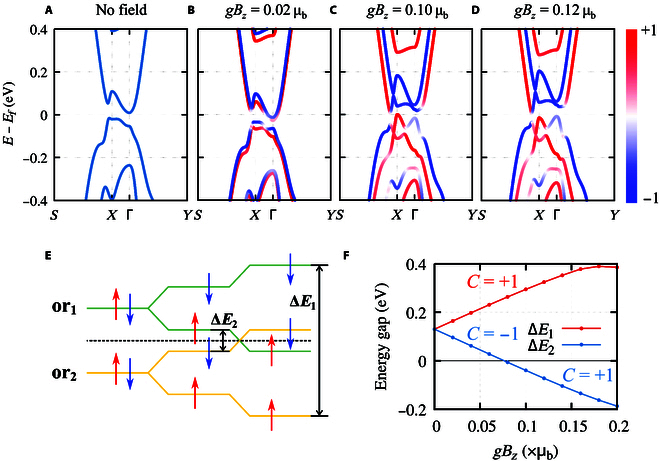
Evolution of electronic band structure and topological numbers of monolayer TaIrTe_4_ under magnetic field. (A) The band structure without magnetic field. (B to D) The spin-resolved band structures under different *gB_z_* values. The red and blue colors represent the *z* component of spin channels. (E) Schematic diagram of the band inversion progress at *X* point with an increasing magnetic field. The green and yellow lines stand for the different orbitals near Fermi energy, namely, or1 and or2, and the red and blue arrows stand for the spin states. Δ*E*_1_ = *E*_or1 − down_ − *E*_or2 − up_ and Δ*E*_2_ = *E*_or1 − up_ − *E*_or2 − down_ are energy differences between the spin-up(down) state of or1 and spin-down(up) state of or2. (F) The energy change of Δ*E*_1_(Δ*E*_2_) at high symmetry point of *X* under an increasing magnetic field *gB_z_*. Δ*E*_1_ states at positive zone in the whole progress, with a constant Chern number 1. Δ*E*_2_ changes sign at around *gB_z_* = 0.08 μ_B_, along with the change of Chern number from −1 to 1.

On the basis of the experimental reported lattice structure [40], the electronic band structures evolution of the monolayer TaIrTe_4_ is calculated with a tuning external magnetic field. As presented in Fig. 3A, under the condition without external field, there is a band inversion at *X* point between the conduction and valence bands with a bandgap of ∼24 meV, which can be seen from the “W shape” of the dispersion near the bottom of conduction bands, in good agreement with previous reports [39,42]. The *Z*_2_ topological feature can be directly confirmed by the Wannier center evolution. From Fig. 3A, one can easily see that the evolution of Wannier centers in *k*_1_ − *k*_2_ plane presents as a zigzag form with changing partners at the time-reversal invariant point. Hence, the evolution lines cross the reference line an odd number of times in the half Brillouin zone (BZ).

**Fig. 4.**
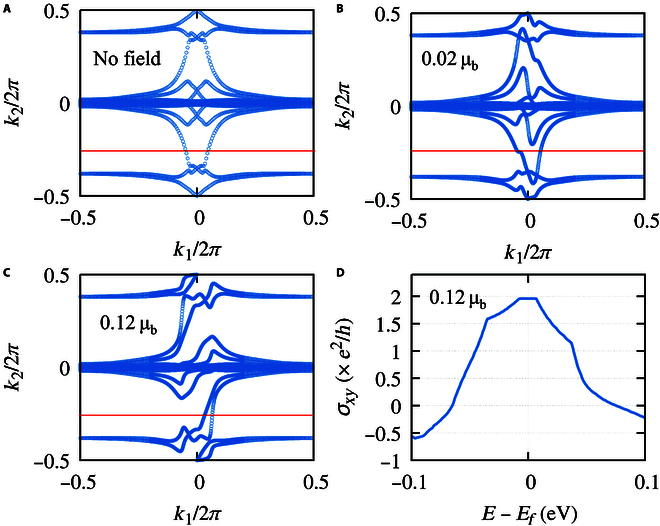
Wannier center evolution in different conditions. (A to C) Wannier center evolution under different magnetic fields. (D) Anomalous Hall conduct of the QH state in the unit of *e*^2^/*h* under different chemical potentials.

The magnetic field serves as an effective way to tune the electronic band structure and band order as it breaks the time-reversal symmetry. We try to apply an external magnetic field perpendicular to the monolayer TaIrTe_4_ (see the sketch in Fig. 1A). As long as a nonzero magnetic field is introduced, the degeneracy between spin-up and spin-down is broken, and an obvious Zeeman split could be observed in both valence and conduction bands (see Fig. 3B). Correspondingly, the applied external magnetic field breaks the time-reversal symmetry of the Wannier center evolution, leading to an imbalance between the positive and negative parts along *k*_1_, as shown in Fig. 4A and B.

As the magnetic field increases, the spin-up and spin-down channels move in opposite directions within energy space, as shown in Fig. 3B to D. In this mechanism, the original band order between orbital1-spin-down and orbital2-spin-up channels remains unchanged, with only an increase in the magnitude of the inverted bandgap. On the contrary, the spin-up channel from orbital1 moves down, while the spin-down channel from orbital2 moves up, which results in a new band inversion between orbital1-spin-up and orbital2-spin-down states, as illustrated in the schematic diagram of Fig. 3E.

From the band-number-indexed Berry phase calculations, we found that the original band inversion due to the crystal field and spin-orbital coupling, i.e., the band inversion between orbital1-spin-down and orbital2-spin-up, hosts a Chern number 1. Similarly, the initial band inversion between orbital1-spin-up and orbital2-spin-down hosts a Chern number −1. Hence, the net Chern number maintains 0 under a weak field. However, as the magnetic field gradually increases, the newly generated band inversion between orbital1-spin-up and orbital2-spin-down has flipped its Chern number from −1 to 1. Consequently, the new phase with magnetic field above 0.1 μ_B_ hosts a net Chern number 2 and a nonzero quantized Hall conductance of 2*e*^2^/*h*.

The evolution of bandgaps between different orbital and spin characters, i.e., Δ*E*_1_ = *E*_or1 − down_ − *E*_or2 − up_ and Δ*E*_2_ = *E*_or1 − up_ − *E*_or2 − down_, is given in Fig. 3F. We can see that the positive bandgap of Δ*E*_2_ decreases to zero at around *gB_z_* = 0.08 μ_B_ and then goes to the negative zone. Correspondingly, the Chern number between orbital1-spin-up and orbital2-spin-down changes from −1 to 1. On the other hand, the bandgap of Δ*E*_1_ follows an opposite trend as it keeps staying at the positive zone with a constant Chern number 1 within a magnetic field of 0.12 μ_B_. Therefore, the topological phase transition is mainly induced by the band order exchange between orbital1-spin-up and orbital2-spin-down. The sign change of Chern number from −1 to 1 for the bandgap of Δ*E*_2_ leads to a net Chern number 2 for the whole system.

The QH insulating state was also confirmed by the Wannier center evolution in the 2D BZ under a magnetic field of *gB_z_* = 0.12 μ_B_. As presented in Fig. 4C, the evolution of the Wannier center in the whole range of *k*_1_ axis is calculated because of the time-reversal symmetry breaking. When fixing the band number *n* as the fully occupied states, the Wannier center evolution lines cross the reference line twice, where both 2 evolution lines show a positive slope. From the energy dispersion in Fig. 3D, a global bandgap around 14 meV is observe, and, thus, a quantized Hall conductance is expected. We then calculated the chemical-potential-dependent Hall conductance by following the linear response Kubo formula (see Methods for details). From Fig. 4D, we can see a stable plateau of 2*e*^2^/*h* near the charge neutrality point at the situation of *gB_z_* = 0.12 μ_B_, fully in agreement with the band-number-indexed Berry phase analysis and Wilson loop calculations. On the basis of the above analysis, we tried to rotate the magnetic field to the −*z* direction and found that both the slope of Wannier center evolution and Hall conductance change the signs.

**Fig. 5.**
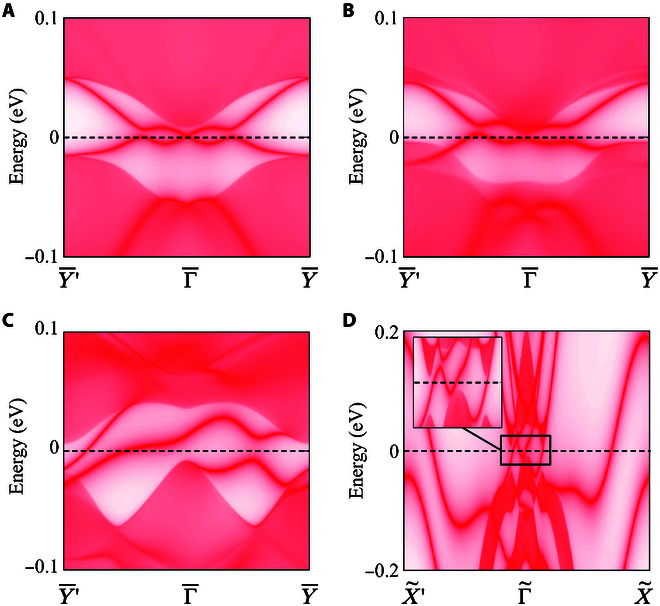
Projected edge states in different situations for the monolayer TaIrTe_4_ with and without magnetic field. (A) [010] surface without magnetic field. (B) [010] surface with *gB_z_* = 0.02 μ_B_. (C) [010] surface with *gB_z_* = 0.12 μ_B_. (D) [100] surface with *gB_z_* = 0.12 μ_B_. The inset in (D) is an enlarged surface states near $\tilde{\Gamma }$. The other edge states away from $\tilde{\Gamma }$ could be attributed to the topological order of intravalance bands.

For both QSH insulators and QH insulators, the quantized signals originate from the edge states located inside the bulk bandgaps. Figure 5A is the projected edge states for the QSH insulator phase along $\bar{Y}-\bar{\Gamma }-\bar{Y}$, with spin helical linear crossing edge Dirac point locating at $\bar{\Gamma }$. As long as a *z*-oriented magnetic field is applied, the Dirac point is broken by opening an anticrossing-like bandgap, due to time-reversal symmetry breaking (see Fig. 5B). After the new band inversion happens between orbital1-spin-up and orbital2-spin-down at *X* point, the spin helical edge states transfer to chiral edge states with positive velocity connecting the occupied and nonoccupied bulk bands (see Fig. 5C and D). Although the specific shapes of the edge states are dependent on the details of edge potentials, the net crossing points between the chiral edge states and Fermi levels are both 2 for the open boundary condition along *x* and *y* directions, fully in consistent with the bulk topological charge analysis. Therefore, the quantized signals are switchable between longitudinal conductance and transversal Hall conductance, via tuning the external magnetic field.

**Fig. 6.**
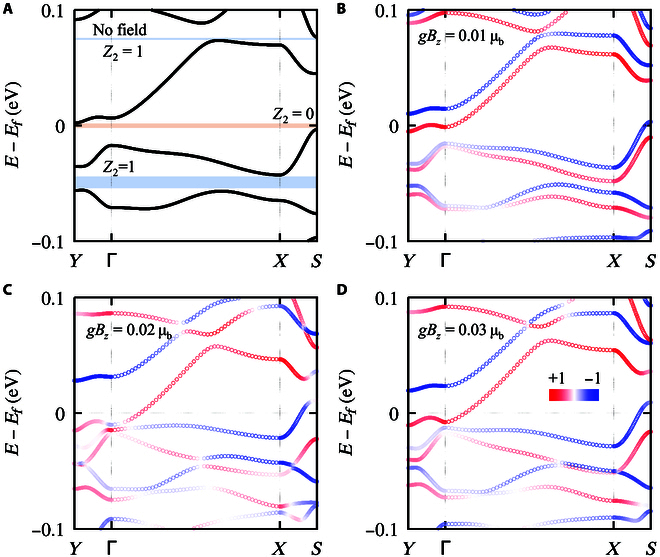
(A) The band structure of the CDW state of TaIrTe_4_ without magnetic field. (B to D) The spin-resolved band structures of the CDW state of TaIrTe_4_ under different *gB_z_* values. The red and blue colors represents the *z* component of spin channels.

In addition to the QSH insulator phase of the primitive cell of the monolayer TaIrTe_4_ at the charge neutrality point, an unconventional CDW phase with 2 nontrivial gaps was also observed by weakly gating [37]. With the inspiration from this experimental result, we also analyzed the electronic structure of this CDW phase under a magnetic field, as shown in Fig. 6. After tuning the superlattice potential to *V* = 0.13 eV, 2 nontrivial gaps appear within a chemical potential of 0.1 eV, as shown in Fig. 6A, in consistent with the previously report [37]. After applying a nonzero magnetic field to the CDW phase, the degeneracy between 2 spin channels is broken, and a Zeeman split is induced, as presented in Fig. 6B to D. As the magnetic field increases, all the valence and conduction bands of these 3 bandgaps gradually get touched. However, all these 3 bandgaps disappear after band crossings, and the CDW phase of TaIrTe_4_ undergos a insulator–metal phase transition within this energy window, different from the phase transition in the primitive cell of monolayer TaIrTe_4_.

## Conclusion

In summary, we studied the magnetic-field-induced topological phase transition in the newly discovered dual QSH insulator TaIrTe_4_. After applying a magnetic field along *z* direction, the original band order of the inverted bandgap from one branch of the spin-up and spin-down channels is exchanged, with the corresponding Chern number transferring from −1 to 1. Together with another original branch of band inversion with Chern number 1, the topological phase transition happens between the QSH insulator and the QH insulator. Since the QSH insulator state in monolayer TaIrTe_4_ can host a nonzero quantized longitudinal conductance that rarely exists in other 2D *Z*_2_ topological insulators, such topological phase transition is along with the exchange of quantized signals between longitudinal conductance and transversal Hall conductance. This result also proposes another effective strategy to verify the existence of QSH insulating state in TaIrTe_4_.

## Methods

Ground-state study of monolayer TaIrTe_4_ and the Wannier projection is carried out in full-potential local-orbital package under generalized gradient approximation with Perdew–Burke–Ernzerhof parametrization [43–45]. Self-consistent energy reaches a convergence of 10^−6^ eV. Structure from the experimental is applied with lattice constants of *a* = 12.42 Å and *b* = 3.77 Å, and a vacuum of 15 Å is applied on *c* axis to eliminate the interlayer interaction. The CDW phase is simulated by a superlattice modulation of Fröhlich–Peierls Hamiltonian of $H=H_{r}+V\text{cos}\left(\mathrm{Qx}\right)u_{r}^{†}u_{r}$, where *V* is the CDW modulation strength, *Q* is the magnitude of the CDW vector, and *x* is the Wannier center coordinate along *x* axis [37,46]. Magnetic field is simulated by adding Zeeman splitting Hamiltonian on Wannier basis *H*_0_ that reads *H* = *H*_0_ + *H_Z_*, where *H_Z_* = *g****B*** · ***σ***. For out-of-plane magnetic field ***B***∥ *z*, it could be written as a scalar *H_Z_* = *gB_z_σ_z_*. In comparison to the spin moment, the effect from orbital moments is relatively small and can be negligible. On the basis of *H*, edge-state calculation is performed by the iteration of green function [47], while the Wannier center evolution is carried out by the Wilson loop method [48]. Anomalous Hall conductivity with varying chemical potential is performed by the Kubo formula [49] in clean limit

σxyE=e2ℏS∫‍dk∑i‍fiEkΩxyikΩxyik=∑j‍Imrxijkryjik (1)

where $r_{a}^{\mathrm{ij}}\left(k\right)=i\langle u_{i}\left(k\right)\;|\;\partial_{k_{a}}\;|\;u_{j}\left(k\right)\rangle$, *u_i_*(***k***) represents the *i*th Wannier state, *S* is the in-plane area of monolayer TaIrTe_4_, and *H_ij_*(***k***) is the Hamiltonian of the system, while *f^i^*(*E*, ***k***) is the occupation of band index *i* with momenta ***k*** under a chemical potential *E*. For the numerical integration over BZ, a *k*-point sampling of 6 × 18 × 1 is applied for the ground-state density functional theory study, while a sampling of 2,000 × 2,000 × 1 is used for the anomalous Hall conductivity calculation.

## Data Availability

The data that support the findings of this study are available from the corresponding author upon reasonable request.
